# Dose and Duration of Upfront Steroid Administration Have no Prognostic Impact in Dogs With Multicentric Diffuse Large B‐Cell Lymphoma

**DOI:** 10.1111/vco.70004

**Published:** 2025-07-08

**Authors:** Ilaria Maga, Silvia Sabattini, Valeria Martini, Fulvio Riondato, Luca Aresu, Laura Marconato

**Affiliations:** ^1^ Department of Veterinary Medical Sciences University of Bologna Bologna Italy; ^2^ Department of Veterinary Medicine University of Milan Lodi Italy; ^3^ Department of Veterinary Sciences University of Turin Turin Italy

**Keywords:** canine, corticosteroids, DLBCL, flow cytometry, prognosis

## Abstract

Steroids provide rapid clinical improvement in dogs with multicentric diffuse large B‐cell lymphoma (DLBCL). However, their use before chemotherapy can induce chemoresistance and compromise diagnostic yield due to increased apoptotic cells. This retrospective study assessed the impact of steroid dose and duration on flow cytometry (FC) diagnostic yield and clinical outcomes in dogs with DLBCL subsequently treated with chemotherapy. Of 273 dogs diagnosed with DLBCL between January 2014 and March 2024, 67 (24.5%) received steroids before treatment (median dose: 1 mg/kg, range: 0.5–3 mg/kg; median duration 8 days, range: 1–1080 days). In 94.0% of cases, steroids were administered for lymphoma management. All dogs received CHOP‐based chemotherapy, and 38 (56.7%) also received immunotherapy. Median time to progression (TTP) and lymphoma‐specific survival (LSS) were 143 days (95% CI: 111–175) and 196 days (95% CI: 152–240), respectively. Steroid dose, duration, and cumulative dose had no significant impact on TTP or LSS. However, the addition of immunotherapy was associated with longer LSS (*p* = 0.023). FC diagnostic yield was lower in steroid‐treated dogs compared to 67 non‐pre‐treated dogs (*p* = 0.042). However, within the pre‐treated group, neither dose nor duration impacted diagnostic yield (*p* > 0.05). In addition, TTP (*p* = 0.003) and LSS (*p* < 0.001) were significantly longer in non‐pre‐treated dogs compared to steroid‐treated dogs. These findings suggest that the detrimental effects of upfront steroids are independent of dose or duration. Given their potential to compromise diagnosis and treatment outcomes, corticosteroids should be used with caution and reserved for cases where clinical benefits clearly outweigh the risks.

## 1 Introduction

Synthetic corticosteroids, such as prednisone, prednisolone, methylprednisolone and dexamethasone, are among the most frequently utilised drug classes in veterinary medicine, particularly in the management of cancer patients. Their widespread use stems from their ability to modulate multiple biochemical and cellular pathways involved in inflammation and immune regulation.

In canine lymphoma, corticosteroids exert a therapeutic effect by inducing apoptosis of lymphoid cells, leading to a rapid reduction in lymph node (LN) size and alleviation of clinical signs [1, 2]. However, a well‐documented concern is their potential to induce chemoresistance when administered before chemotherapy initiation [3, 4, 5, 6]. This resistance, which adversely affects prognosis, is linked to the activation of P‐glycoprotein, a drug‐efflux pump that reduces intracellular concentrations of chemotherapy agents, including glucocorticoids themselves [7, 8]. Prolonged or high‐dose corticosteroid therapy exacerbates this effect, as the induction of apoptosis in steroid‐sensitive tumour cell populations leaves behind a more resistant clone, reducing the efficacy of subsequent chemotherapeutic regimens [9, 10]. Beyond chemoresistance, corticosteroids can also interfere with diagnostic procedures. Indeed, a high rate of apoptosis and cell death, regardless of the underlying cause, is a well‐recognised factor contributing to non‐diagnostic cytology samples and inconclusive flow cytometry (FC) results, potentially leading to treatment delays [11]. Thus, in suspected lymphoma cases, it is strongly recommended to postpone corticosteroid administration until a definitive diagnosis is obtained and treatment options, including their impact on prognosis, have been discussed with the owner. For these reasons, corticosteroids should be prescribed with caution and with a thorough understanding of their pharmacologic benefits and limitations, ensuring that they are introduced only when the expected clinical benefits outweigh the potential risks. While multiple studies have demonstrated the negative impact of steroid exposure on lymphoma treatment outcomes [3, 4, 5, 6], the extent to which dosage and duration of administration before chemotherapy influence this effect remains unclear. Moreover, these studies involved a heterogeneous population of dogs with lymphoma, without stratification by histotype or clinical stage [3, 4, 5, 6]. This variability likely affected both treatment response and survival outcomes, thereby limiting the ability to accurately assess the specific impact of pre‐treatment corticosteroid administration on individual lymphoma subtypes.

The aim of this single‐centre retrospective study was to gather detailed information on the dose and duration of steroid administration in dogs with multicentric diffuse large B‐cell lymphoma (DLBCL) that subsequently received chemotherapy, and to evaluate its impact on both the diagnostic yield of FC and clinical outcomes. In addition, the clinical outcomes of dogs that received upfront steroids were compared to those of dogs that did not.

## 2 Methods

### 2.1 Study Design

A retrospective review of the hospital's electronic medical records from the Oncology Unit of the Department of Veterinary Medical Sciences at the University of Bologna, Italy, was conducted to identify dogs newly diagnosed with lymphoma from January 2014 to March 2024. Dogs were ultimately included in the study if they had a histopathologic diagnosis of DLBCL and had received corticosteroids within the 30 days preceding chemotherapy administration.

As this evaluation did not influence any therapeutic decision, approval by an Ethics Committee was not required. Owners gave their written informed consent to the use of clinical data. All the procedures were performed in accordance with the Italian legislation for animal welfare (DL March 14, 2014, n. 26) and the best standard for veterinary practice (Good Veterinary Practice from the Veterinary Italian National Federation of the Veterinary Orders January 29, 2005).

### 2.2 Diagnosis and Initial Staging

For all included dogs, the initial assessment consisted of history and physical examination, followed by staging procedures. These included complete blood count with differential, serum biochemistry profile, urinalysis, thoracic radiographs, abdominal ultrasound, cytologic evaluation of liver and spleen regardless of their ultrasonographic appearance and immunophenotype determined by FC on a LN aspirate, peripheral blood (PB) and bone marrow (BM) aspirate. In all dogs, lymphadenectomy of a peripheral enlarged LN was performed to confirm the diagnosis, according to WHO guidelines [12].

### 2.3 Details of Steroid Administration

For each dog, data regarding steroid administration were collected, including the duration of administration, the number of days between the last steroid administration and clinical diagnosis, the type of steroids, daily dose in mg/kg, and clinical indication (treatment of lymphoma or non‐cancer‐related comorbidities). Given the varying relative potencies of glucocorticoids, a dose conversion was performed to standardise the daily doses of methylprednisolone and dexamethasone into mg/kg equivalent of prednisone/prednisolone. For reference, prednisone/prednisolone is approximately five times as potent as cortisol, methylprednisolone is about four times as potent and dexamethasone is approximately 25 times as potent [13]. For each included dog, the cumulative dose of corticosteroids was defined as the total amount administered over a specified period, calculated by multiplying the daily dose (mg/kg) by the patient's body weight (kg) and the number of treatment days. Dogs without detailed information regarding steroid dosage and schedule were excluded from the study.

### 2.4 Treatment and Outcome Assessment

For all dogs enrolled in the study, the treatment regimen included a 12‐week CHOP‐based protocol with or without active immunotherapy, consisting of an autologous vaccine composed of hydroxylapatite ceramic powder and heat shock proteins purified from the dogs' involved LNs [14]. The CHOP protocol included vincristine, cyclophosphamide and doxorubicin over a 12‐week period [15], and was initiated on the day of diagnosis in all cases. Treatment response was evaluated at each treatment session according to previously published criteria [16]. Responses were required to last for at least 28 days. After completion of treatment, a regular monthly follow‐up was required for the first year, and every 2 months thereafter. In case of progression or relapse, rescue treatment was offered to all owners.

For assessing the effect of steroids on outcome, the primary endpoints were response to CHOP and time‐to‐progression (TTP). The secondary endpoint was lymphoma‐specific survival (LSS).

### 2.5 Diagnostic Yield by FC

Samples for FC were processed at the FC facilities of the Veterinary Teaching Hospital of the University of Milan or the University of Turin, following previously published protocols [17]. The antibody panel and fluorochrome combinations varied over time and between facilities. However, a minimum panel was consistently applied to all samples, comprising the following antibodies: CD45 (clone YKIX716.13), CD5 (clone YKIX322.3), CD21 (clone CA2.1D6), and CD34 (clone 1H6). For each case, a corresponding LN cytologic specimen was also assessed by the same operator who processed the FC sample and was subjectively evaluated for compatibility with large cell lymphoma.

To assess the impact of pre‐treatment with corticosteroids on the diagnostic yield, a control group was established for comparison. This group included dogs with histologically proven DLBCL that had not received corticosteroids within 30 days before diagnosis, and their FC analyses were conducted at the same facilities and during the same timeframe. An equal number of dogs was included in the control and pre‐treated groups.

To ensure a standardised assessment of diagnostic yield and minimise inter‐observer variability, raw FC data were retrieved and re‐analysed through a consensus between two experienced flow cytometrists (F.R. and V.M.), who were blinded to any history of corticosteroid pre‐treatment. Cases without available FC data for review were excluded from this study.

Following the review, cases were categorised into the following groups: Group 1, LN aspirates diagnostic by FC for large B‐cell lymphoma, characterised by the presence of > 20% large B cells [17]; Group 2, LN aspirates not diagnostic for lymphoma by FC, but cytology findings compatible with large cell lymphoma (as reviewed at the time of data analysis) and concomitant evidence of PB or BM infiltration by large B cells (> 0.56% and > 2.45% large B cells, respectively) [18]; Group 3, LN aspirates not diagnostic for lymphoma by FC and without PB or BM infiltration by large B cells. LN aspirates were deemed non‐diagnostic by FC if they contained < 20% large B cells or were not processed due to gross artefacts or poor cellularity, based on the operator's assessment at the time of diagnosis. LN samples from both Groups 2 and 3 were non‐diagnostic by FC. In Group 2, the diagnosis was still achievable based on high‐quality cytology and evidence of PB or BM infiltration, as previously described [11].

### 2.6 Statistical Analysis

Descriptive statistics were used in the analysis of dogs and tumour characteristics. When appropriate, data sets were tested for normality by use of the D'Agostino and Pearson omnibus normality test. Since none of the data followed a normal distribution, results were expressed as median (range).

For dogs pre‐treated with steroids, the recorded data included demographics (breed, sex, age, weight), stage and substage according to WHO, presence of extranodal involvement, presence of haematologic abnormalities (anaemia, low platelet count, elevated LDH, hypercalcemia), date of diagnosis, details of steroid administration, treatment protocol (chemotherapy vs. chemo‐immunotherapy), treatment response [16], date of progression, date and cause of death.

TTP was defined as the interval between the initiation of chemotherapy and tumour progression. LSS was defined as the interval between the initiation of chemotherapy and lymphoma‐related death. To compare outcomes between dogs pre‐treated with steroids and the control group of dogs not pre‐treated with steroids, TTP and LSS were also calculated from the beginning of steroid administration. Dogs that did not experience tumour progression or lymphoma‐related death were censored at the time of their last follow‐up. Survival estimates were reported as medians with 95% confidence intervals (95% CIs). In the group of dogs pre‐treated with steroids, the impact of haematologic abnormalities, stage, substage, extranodal involvement, haematologic abnormalities, steroid dosage, duration of steroid administration, cumulative dose and treatment protocol on TTP and LSS was assessed using Cox regression analysis. Variables that were significant (*p* < 0.05) in the univariable analysis were included in the multivariable model. A univariable Cox regression analysis was also performed to compare outcomes between dogs pre‐treated with steroids and the control group of non‐pre‐treated dogs. For this analysis, TTP and LSS were also calculated from the initiation of steroid therapy.

The impact of steroid dosage, duration of administration and cumulative dose on the response to the CHOP protocol was analysed using the Pearson chi‐squared test or Fisher's exact test, depending on the expected frequencies in contingency tables. To ensure meaningful comparisons, steroid‐related variables were categorised using specific thresholds based on the available sample size and data distribution. In particular, the duration of administration was categorised as < 10 or ≥ 10 days, the daily dose as < 1 or ≥ 1 mg/kg, and the cumulative dose was dichotomised using the median as the cutoff. Differences in CHOP protocol response between the pre‐treated and non‐pre‐treated dogs were also assessed using Pearson's chi‐squared test or Fisher's exact test, as appropriate. To evaluate potential differences in FC diagnostic yield between pre‐treated dogs and controls, contingency tables were constructed, and a Pearson chi‐squared test was performed. In addition, a multinomial logistic regression was conducted within the pre‐treated group to determine whether the daily dose or cumulative dose varied among FC groups.

Statistical analysis was performed using SPSS Statistics v.25 (IBM, Armonk, NY, USA). Significance was set at *p* < 0.05.

### 2.7 Cell Line Validation Statement

No cell lines were used in the current study.

## 3 Results

Within the indicated inclusion period, 273 (75.5%) dogs were newly diagnosed with multicentric DLBCL. Among them, 206 dogs had not received steroids before chemotherapy, while 67 (24.5%) had.

### 3.1 Dogs Not Pre‐Treated With Steroids

Fifty‐four (26.2%) dogs were mixed breed, while 152 (73.8%) were purebred, with German shepherd (*n* = 20; 13.2%), Rottweiler (*n* = 15; 9.9%), Dobermann (*n* = 9; 5.9%), Golden retriever (*n* = 9; 5.9%), Labrador retriever (*n* = 8; 5.3%) and Bernese Mountain dog (*n* = 8; 5.3%) being the most frequent breeds. There were 110 (53.4%) females (80 spayed) and 96 (46.6%) males (27 neutered). The median age was 8 years (range: 2–16) and median weight was 28.8 kg (range: 2.6–81.3).

Regarding laboratory analysis, 109 (52.9%) dogs had increased LDH activity, 27 (13.1%) dogs were thrombocytopenic, 18 (8.7%) dogs were anaemic and 1 (0.5%) had increased ionised calcium.

At admission, 150 (72.8%) dogs were asymptomatic and 56 (27.2%) showed clinical signs.

One (0.5%) dog had Stage I disease (substage a), 6 (2.9%) dogs had Stage III disease (all substage a), 49 (23.8%) had Stage IV disease (38 substage a, 11 substage b), and 150 (72.8%) had Stage V disease (105 substage a, 45 substage b).

Overall, 36 (17.5%) dogs had extranodal involvement, including lung (*n* = 23), eyes (*n* = 4), intestine (*n* = 2), skin (*n* = 2), peritoneal malignant effusion (*n* = 1), kidney (*n* = 1), mammary gland (*n* = 1), lung and eye (*n* = 1), lung and peritoneal effusion (*n* = 1). Lymphoma was confirmed by cytology in dogs with intestinal, cutaneous, renal and mammary involvement or malignant effusion, whereas in the remaining cases, the diagnosis was presumed based on physical examination and imaging findings.

All dogs were treated with the same CHOP protocol. In addition, 2 (1%) dogs received l‐asparaginase as part of their treatment. One hundred seventeen (56.8%) dogs also received immunotherapy [14].

### 3.2 Dogs Pre‐Treated With Steroids

Twenty (29.9%) dogs were mixed breed, while 47 (70.1%) were purebred, with French bulldog (*n* = 5; 10.6%), Rottweiler (*n* = 4; 8.5%), Dobermann (*n* = 3; 6.4%) and Labrador retriever (*n* = 3; 6.4%) being the most frequent breeds. There were 37 (55.2%) males (6 neutered) and 30 (44.8%) females (17 spayed). The median age was 7 years (range: 3–15) and the median weight was 24.1 kg (range: 4–67.1).

Regarding laboratory analysis, 34 (50.7%) dogs had increased LDH activity, 14 (20.9%) dogs were thrombocytopenic, 9 (13.4%) dogs were anaemic and 2 (2.9%) had increased ionised calcium.

Based on feedback from owners or referring veterinarians, 36 (53.7%) dogs were classified as asymptomatic (substage a) before starting steroids, while 31 (46.3%) were considered symptomatic (substage b). Three (4.5%) dogs had Stage III disease (all substage a), 15 (22.4%) had Stage IV disease (13 substage a, 3 substage b) and 49 (73.1%) dogs had Stage V disease (20 substage a, 29 substage b). Overall, 19 (28.4%) dogs had extranodal involvement, including lung (*n* = 10), eyes (*n* = 4), intestine (*n* = 3), peritoneal malignant effusion (*n* = 1), eyes and lung (*n* = 1). Lymphoma was confirmed by cytology in dogs with intestinal involvement or malignant effusion, whereas in the remaining cases, the diagnosis was presumed based on physical examination and imaging findings.

The median duration of steroid treatment was 8 days (range: 1–1080), with a median dose of 1 mg/kg (range: 0.5–3). The median cumulative dose was 198 mg (range: 11.8–10 800). The steroids prescribed included prednisone (*n* = 57; 85.0%), prednisolone (*n* = 6; 9.0%), methylprednisolone (*n* = 3; 4.5%) and dexamethasone (*n* = 1; 1.5%). In 63 (94.0%) cases, steroids were administered for lymphoma management. In the remaining cases, they were prescribed for dermatitis (*n* = 1; 1.5%), uveitis (*n* = 1; 1.5%), suspected otitis (*n* = 1; 1.5%) and suspected tracheitis (*n* = 1; 1.5%). The median time between steroid discontinuation and both FC analysis and chemotherapy initiation was 1 day (range: 0–2).

All dogs were treated with the same CHOP protocol. In addition, three (4.5%) dogs received l‐asparaginase as part of their treatment. Thirty‐eight (56.7%) dogs also received immunotherapy [14].

### 3.3 Treatment Response and Outcome

Among dogs not pre‐treated with steroids, 168 (81.6%) achieved complete remission (CR) and 30 achieved partial remission (PR). Four (1.9%) dogs experienced stable disease (SD), while four (1.9%) dogs progressed.

Among the 198 dogs that achieved CR or PR, 147 (74.2%) experienced at least one relapse, with 42 of them having multiple relapses.

Rescue treatments were administered to 126 dogs, utilising the following protocols: CHOP rechallenge (*n* = 80), CHOP followed by DMAC (dexamethasone, melphalan, actinomycin‐D and cytarabine; *n* = 14), DMAC (*n* = 8), lomustine (*n* = 8), CHOP followed by LOPP (lomustine, vincristine, procarbazine and prednisone; *n* = 5), prednisone (*n* = 3), l‐asparaginase and lomustine (*n* = 2), l‐asparaginase (*n* = 2), LOPP (*n* = 2), CHOP followed by PPC (procarbazine, prednisone, cyclophosphamide; *n* = 1), PPC (*n* = 1), CHOP followed by lomustine (*n* = 1), CHOP followed by lomustine and DMAC (*n* = 1).

**FIGURE 1:**
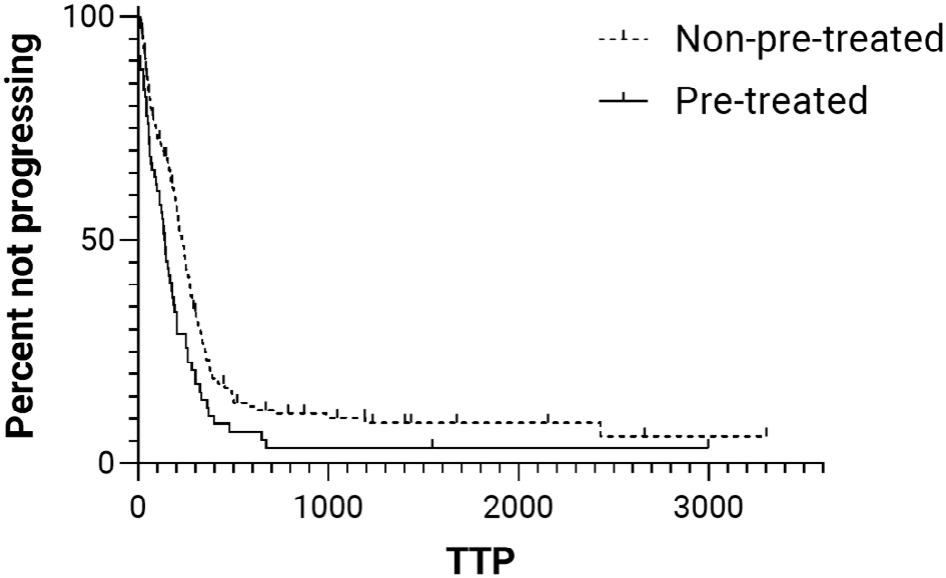
Kaplan–Meier curves of time to progression (TTP) for dogs with multicentric diffuse large B‐cell lymphoma pre‐treated (solid line; median, 143 days) or non‐pre‐treated (dashed line; median, 223 days) with steroids (HR = 1.6, 95% CI = 1.2–2.1; *p* = 0.003). Tick marks represent censored cases.

**FIGURE 2:**
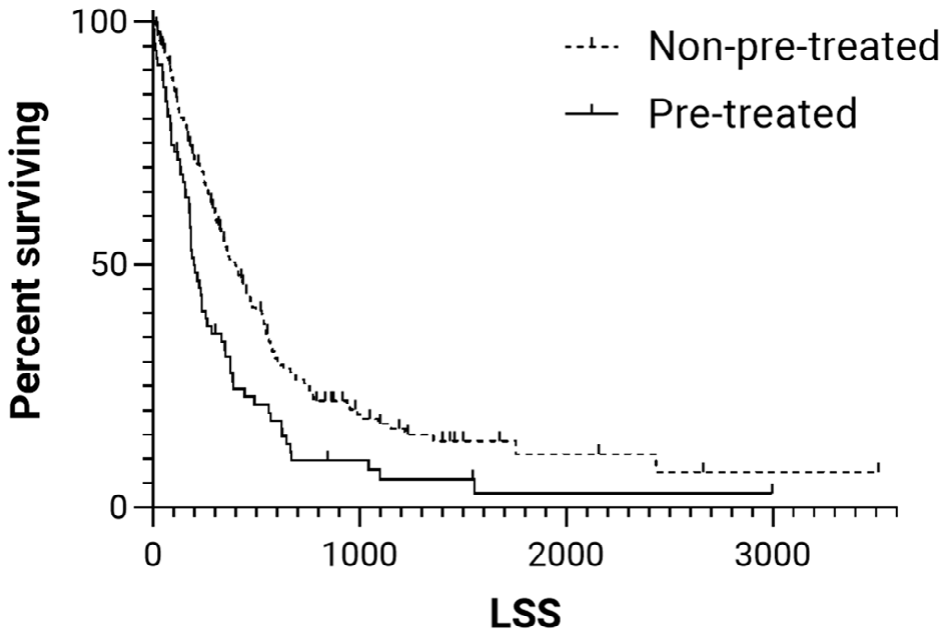
Kaplan–Meier curves of lymphoma‐specific survival (LSS) for dogs with multicentric diffuse large B‐cell lymphoma pre‐treated (solid line; median, 196 days) or non‐pre‐treated (dashed line; median, 398 days) with steroids (HR = 1.7, 95% CI = 1.3–2.3; *p* < 0.001). Tick marks represent censored cases.

The median TTP was 223 days (95% CI: 206–260 days; Figure 1). At the time of writing, 30 (14.6%) dogs were still alive, with a median follow‐up period of 490 days. The remaining 176 patients have died, 142 (80.7%) due to lymphoma‐related causes. The median LSS was 398 days (95% CI: 328–468 days; Figure 2).

Among pre‐treated dogs, 45 (67.2%) achieved CR and 11 (16.4%) achieved PR. One (1.5%) dog experienced SD, while 10 (14.9%) dogs progressed. Among the dogs that achieved CR or PR, 50 (89.3%) experienced at least one relapse, with 12 of them having multiple relapses. Rescue treatments were administered to 33 dogs, utilising the following protocols: CHOP rechallenge (*n* = 22), CHOP followed by lomustine (*n* = 3), lomustine (*n* = 3), CHOP followed by DMAC (*n* = 2), DMAC (*n* = 1), verdinexor (*n* = 1) and l‐asparaginase (*n* = 1).

The median TTP was 143 days (95% CI: 111–175 days; Figure 1). At the time of writing, only one patient was still alive, with a follow‐up period of 2996 days. The remaining patients have died, 61 (91%) due to lymphoma‐related causes. The median LSS was 196 days (95% CI: 152–240 days; Figure 2).

On univariable analysis, no variable was significantly associated with tumour progression, whereas anaemia, Stage V disease, substage b, extranodal involvement, and lack of immunotherapy administration were significantly associated with lymphoma‐related death (Table 1).

**TABLE 1:** Univariable Cox regression analysis of variables potentially associated with increased risk of tumour progression and disease‐related death in 67 dogs with multicentric diffuse large B‐cell lymphoma pre‐treated with steroids.

Variable	No. of dogs (%)	Tumour progression		Lymphoma‐related death	
		Hazard ratio (95% CI)	*p*	Hazard ratio (95% CI)	*p*
Anaemia	9 (13.4%)	1.7 (0.8–3.7)	0.157	2.2 (1.1–4.6)	0.029*
Low platelet count	14 (20.9%)	1.0 (0.5–1.8)	0.923	0.9 (0.5–1.7)	0.707
Elevated LDH	34 (50.7%)	1.6 (1.0–2.6)	0.008	1.5 (0.9–2.5)	0.133
Hypercalcemia	2 (3.0%)	1.5 (0.4–6.1)	0.597	2.1 (0.5–8.9)	0.305
Stage V	49 (73.1%)	1.7 (0.9–3.2)	0.080	2.0 (1.0–3.7)	0.035*
Substage b	31 (46.3%)	1.5 (0.9–2.5)	0.119	2.0 (1.2–3.4)	0.010*
Extranodal involvement	19 (28.4%)	1.3 (0.8–2.3)	0.316	1.9 (1.0–3.3)	0.024*
Steroid dosage ≥ 1 mg/kg	49 (73.1%)	1.2 (0.7–2.1)	0.501	1.1 (0.6–2.0)	0.655
Steroid duration ≥ 10 days	33 (49.3%)	1.2 (0.7–2.0)	0.482	1.1 (0.7–1.9)	0.668
Steroid cumulative dose ≥ 198 mg	35 (52.2%)	1.1 (0.7–1.8)	0.670	1.1 (0.7–1.9)	0.594
Lack of immunotherapy	29 (43.3%)	1.6 (0.9–2.7)	0.057	2.0 (1.2–3.3)	0.008*

Abbreviation: CI, confidence interval.

* Significant.

On multivariable analysis, only lack of immunotherapy retained prognostic significance (HR: 1.9; 95% CI: 1.1–3.4; *p* = 0.023; Table 2). For dogs treated with CHOP alone, the median LSS was 145 days (95% CI: 54–236), while for those also receiving immunotherapy, it was 263 days (95% CI: 163–360).

**TABLE 2:** Multivariable Cox regression analysis for risk of tumour‐related death in 67 dogs with multicentric diffuse large B‐cell lymphoma pre‐treated with steroids.

Variable	Lymphoma‐related death	
	Hazard ratio (95% CI)	*p*
Anaemia	2.2 (0.9–5.0)	0.064
Stage V	1.3 (0.6–2.6)	0.538
Substage b	1.5 (0.7–2.9)	0.304
Extranodal involvement	1.3 (0.6–2.7)	0.479
Lack of immunotherapy	1.9 (1.1–3.4)	0.023*

*Note:* Variables with a significance level of *P* < 0.05 at univariable analysis were included in the model.

Abbreviation: CI, confidence interval.

* Significant.

All dogs receiving steroids at doses below 1 mg/kg achieved a response (CR, *n* = 15; PR, *n* = 3), compared to 78% of dogs receiving doses equal to or above 1 mg/kg. However, this difference did not reach statistical significance (*p* = 0.056).

The dose of steroid administration did not significantly impact the risk of tumour progression or tumour‐related death.

The duration of steroid administration in responders (median: 7 days; range: 1–130) was not significantly different from that observed in non‐responders (median: 14 days; range: 1–1080; *p* = 0.23). The duration of steroid administration did not influence TTP or LSS, regardless of treatment (chemotherapy vs. chemo‐immunotherapy). Among dogs receiving steroids at doses equal to or above 1 mg/kg for at least 10 days (*n* = 20), the percentage of non‐responders (35%) was significantly (*p* = 0.012) higher compared to the remaining dogs (*n* = 47; 8.5%). However, TTP and LSS did not differ significantly between the two groups. The cumulative dose of steroids had no impact on tumour response, TTP or LSS.

When comparing pre‐treated with not pre‐treated dogs, the risk of tumour progression was significantly higher in pre‐treated dogs, whether calculated from the beginning of CHOP (HR: 1.6; 95% CI: 1.2–2.1; *p* = 0.003; Figure 1) or from the beginning of steroid administration (HR: 1.4; 95% CI: 1–1.8; *p* = 0.036). The same was observed for LSS, with a significantly higher risk of lymphoma‐related death in pre‐treated dogs both when calculated from the start of CHOP (HR: 1.7; 95% CI: 1.3–2.3; *p* < 0.001; Figure 2) and from the start of steroid therapy (HR: 1.6; 95% CI: 1.2–2.1; *p* = 0.004).

The proportion of dogs responding to the CHOP protocol was significantly higher in the dogs not pre‐treated with corticosteroids compared to the pre‐treated group (*p* = 0.013).

#### 3.3.1 Diagnostic Yield by FC and Histology

**FIGURE 3:**
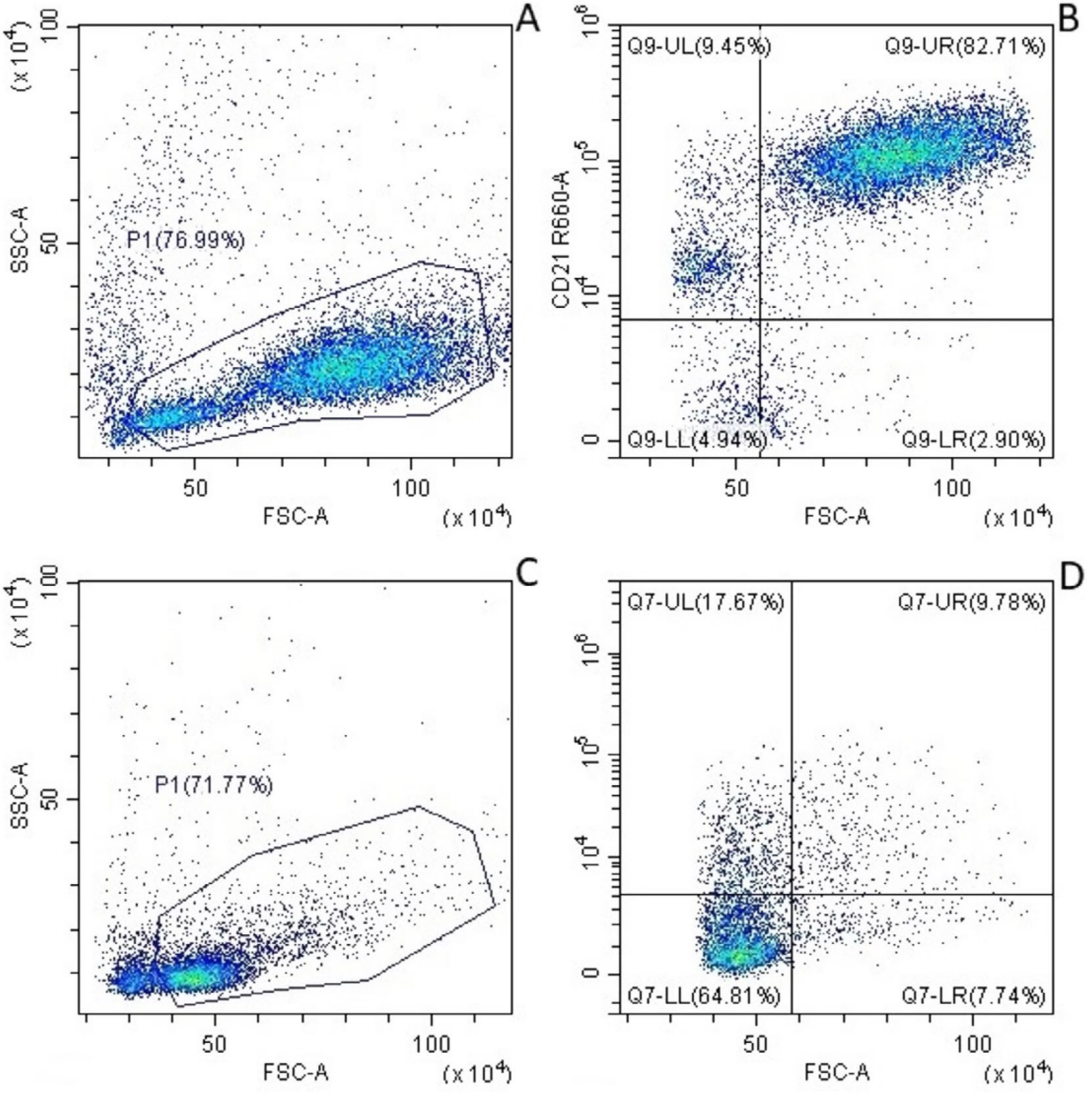
Flow cytometric scattergrams of nodal aspirates obtained from two dogs. (A, B) Sample diagnostic for large B‐cell lymphoma. A population of large cells is identifiable in the morphologic scattergram (A), with more than 20% of large cells staining positive for CD21 (B). (C, D) Sample not diagnostic for large B‐cell lymphoma. No large cells are identified in the morphologic scattergram (C), and large cells staining positive for CD21 account for less than 20% (D).

Overall, 67 pre‐treated dogs and 67 controls were included in the analysis. Among pre‐treated dogs, samples from 58 (86.6%) cases were diagnostic by FC (Group 1; Figure 3A,B), while 3 (4.5%) cases were classified in Group 2 (Figure 3C,D). In addition, 6 (9.0%) cases were deemed non‐diagnostic by FC (Group 3) due to a low number of intact cells and/or the loss of the neoplastic component, with only residual non‐neoplastic lymphocytes detected in FC analysis. In contrast, all samples from the control group were diagnostic by FC, with 63 cases (94.0%) in Group 1 and 4 (6.0%) in Group 2. Although not statistically significant, the proportion of LN aspirates deemed of insufficient quality (Groups 2 and 3 combined) was approximately twice as high in pre‐treated dogs compared to non‐pre‐treated dogs (13.5% vs. 6%, respectively).

The prevalence of non‐diagnostic samples by FC (Group 3) differed significantly between pre‐treated dogs and controls (*p* = 0.042). However, within the pre‐treated group, neither the steroid dose, duration of administration, nor cumulative dose had an impact on diagnostic yield (*p* > 0.05 for all analyses).

Histologic evaluation was conducted on surgically excised LNs a few days after the diagnosis of lymphoma had been confirmed by cytology and FC. Although the dogs had received corticosteroid treatment before biopsy, no significant morphologic alterations were noted compared to cases without prior steroid exposure. Tissue preservation was adequate, and the histologic features were consistent with a classical DLBCL [19].

## 4 Discussion

Current evidence suggests that dogs with aggressive lymphoma receiving glucocorticoids before the initiation of chemotherapy may experience reduced survival. [3, 4, 5, 6] This detrimental effect was also evident in the current cohort of dogs with DLBCL, as those receiving upfront steroids experienced shorter TTP and LSS and lower response rates compared to dogs that did not receive pre‐treatment.

This finding was not unexpected and aligned with previous research [3, 4, 5, 6, 20, 21, 22]. However, our primary interest was to investigate whether the negative impact on prognosis persisted regardless of the steroid dose and duration, aiming to assess whether even minimal corticosteroid exposure adversely affected TTP and LSS. Understanding their impact on treatment outcomes is essential for optimising therapeutic strategies. To investigate this, we evaluated the outcomes of 67 dogs with DLBCL that received corticosteroids before CHOP‐based chemotherapy to determine whether the dose or duration of administration influenced prognosis.

Our findings suggest that steroid dose, duration and cumulative dose had no significant impact on tumour progression or survival. Although all dogs receiving steroids at doses below 1 mg/kg achieved a response, compared to 78% of dogs receiving doses equal to or above 1 mg/kg, this difference did not reach statistical significance.

When evaluating the impact of steroid duration, among dogs that received steroids at doses equal to or above 1 mg/kg for at least 10 days, the percentage of non‐responders (35%) was significantly higher compared to the remaining dogs (8.5%). Despite this difference in initial response rates, there was no significant variation in overall clinical progression between the two groups.

Across all dosing regimens, the duration of upfront steroid administration, which ranged from 1 to 1080 days in this cohort, did not significantly influence outcome. A previous study reported that resistance can develop in as little as 7 days [9]. This aligns with our findings, where the median duration of steroid treatment was 8 days, suggesting that even short‐term administration may have irreversible detrimental effects on prognosis.

A concerning finding was that nearly one‐quarter of the 273 dogs with DLBCL referred to our hospital received corticosteroids before a confirmed diagnosis and before the initiation of chemotherapy. We investigated the reasons for corticosteroid administration and found that the vast majority (94%) of dogs received steroids specifically for lymphoma treatment, while only 6% were treated for unrelated conditions. The use of corticosteroids before chemotherapy is often justified by the need to manage severe clinical signs; however, in the current cohort, only 31 out of 67 dogs (46.3%) presented with substage b disease. Moreover, a review of medical records did not consistently indicate whether the clinical condition was severe enough to justify the initiation of corticosteroid therapy. A substantial proportion of dogs (53.7%) were asymptomatic (substage a) yet still received steroids. This suggests that, in many cases, corticosteroid administration was driven primarily by the lymphoma diagnosis itself rather than by clinical necessity. General practitioners, who are often the first to manage these patients, should be fully aware of the significant risks associated with the administration of corticosteroids before a definitive diagnosis is established. Increased awareness is essential to ensuring optimal management and prognosis for dogs with suspected lymphoma.

Among all evaluated prognostic factors, only the lack of immunotherapy was significantly associated with lymphoma‐related death. While immunotherapy may mitigate the negative impact of upfront steroids and contribute to better survival outcomes, the limited cohort size may have reduced the statistical power to detect significant differences for other prognostic variables, even if clinically relevant.

A second aim of this study was to assess the effect of upfront corticosteroid administration on the diagnostic yield of FC. Sample characteristics, particularly cellularity and necrosis, are key determinants of the likelihood of obtaining a diagnostic sample via FC [11]. Steroid‐induced apoptosis compromises sample quality by reducing the viability of neoplastic cells, thereby hindering accurate FC analysis. Compared to dogs that did not receive steroids before diagnosis, those that did exhibited a reduced diagnostic yield. In detail, 9% of pre‐treated dogs had non‐diagnostic LN results using FC, and in 4.5% of cases, a diagnosis was only possible due to the presence of PB and/or BM infiltration. Had these additional matrices not been evaluated, the proportion of cases for which a diagnosis could not be established would have risen to 13.5% among pre‐treated dogs, compared to 6% in non‐pre‐treated dogs. Clearly, in a proportion of dogs receiving upfront steroids, accurate lymphoma classification and staging are compromised, complicating treatment decisions and potentially delaying the initiation of optimal therapy. In addition, our results support the analysis of all three matrices in every case to maximise the likelihood of obtaining a diagnosis, as previously reported in the literature [11]. By contrast, histopathology followed by immunohistochemistry allowed a definitive diagnosis to be reached in all cases, regardless of prior steroid treatment. This highlights the critical role of this combined approach, especially in diagnostically challenging cases. In situations where cytology or other less invasive techniques may yield inconclusive or misleading results, histology with immunohistochemistry remains the only reliable method to achieve an accurate and conclusive diagnosis.

There are several limitations to be acknowledged, mainly related to the retrospective nature of the study. Although staging procedures, including histologic confirmation, were standardised, treatment protocols varied, with more than half of the dogs receiving immunotherapy alongside CHOP‐based chemotherapy. The decision to incorporate the vaccine was primarily influenced by financial considerations. While immunotherapy may affect TTP and LSS, it did not impact remission rates, as the vaccine is administered after the fourth chemotherapy session, whereas response rates are assessed earlier. While the rationale for upfront steroid administration was examined, objectively assessing the severity of clinical signs that might have warranted its use proved challenging.

In addition, the presence of clinical signs before corticosteroid administration could not be definitively ascertained at the time of admission, as information was based on the owners' retrospective accounts when prompted to explain the reasons for seeking veterinary care. To mitigate this limitation, targeted questions were asked during the clinical interview to help clarify whether the signs reported were systemic and not solely attributable to lymphadenomegaly. We acknowledge that recall bias cannot be entirely excluded, as owners may have had difficulty accurately recalling the timing or nature of the initial symptoms. Nevertheless, this structured approach aimed to reduce misclassification and ensure a more reliable characterisation of pre‐treatment clinical presentation. Last, dogs receiving steroid doses below 1 mg/kg showed a statistical trend towards achieving a response (complete or partial); however, this did not translate into a significant improvement in TTP or LSS. The limited number of dogs treated with low‐dose steroids restricts definitive conclusions. Further studies with a larger population are needed to better evaluate the potential impact of steroid dosage on chemotherapy response. Finally, these findings pertain specifically to DLBCL cases, and it remains unclear whether they are applicable to other histotypes.

In conclusion, glucocorticoid administration before chemotherapy negatively impacts prognosis, irrespective of dosage or duration, by compromising treatment response and overall outcome. Moreover, it can reduce FC diagnostic accuracy, potentially leading to misclassification or delays in appropriate therapy. These findings underscore the importance of carefully evaluating the necessity of corticosteroid use before chemotherapy in dogs with multicentric DLBCL. Future studies should explore whether specific patient subsets might still benefit from short‐term steroid use for symptom management and investigate strategies to minimise its detrimental effects on chemotherapy efficacy.

## Ethics Statement

In compliance with local legislation, ethical approval was not required for this study. Dogs were treated according to the current standards. All owners signed a written informed consent.

## Conflicts of Interest

The authors declare no conflicts of interest.

## Data Availability

The data that support the findings of this study are available on request from the corresponding author.
